# *catena*-Poly[[(diaqua­zinc)-μ-3-carb­oxy­pyrazine-2-carboxyl­ato-κ^4^ *N* ^1^,*O* ^2^;*N* ^4^,*O* ^3^] nitrate]

**DOI:** 10.1107/S1600536811054031

**Published:** 2011-12-21

**Authors:** Wojciech Starosta, Janusz Leciejewicz

**Affiliations:** a Institute of Nuclear Chemistry and Technology, ul. Dorodna 16, 03-195 Warszawa, Poland

## Abstract

The crystal structure of the title compound, {[Zn(C_6_H_3_N_2_O_4_)(H_2_O)_2_]NO_3_}_*n*_, is built of zigzag cationic chains propagating in [010] with nitrate anions located in the space between the chains. The Zn^II^ ion is coordinated by N and O atoms of two symmetry-related ligands in equatorial sites, and by two water O atoms at the axial sites of a distorted octa­hedron. One carboxyl­ate group of the ligand remains protonated, serving as a donor in a short intra­molecular O—H⋯O hydrogen bond. The coordinated water mol­ecules are donors and the nitrate O atoms act as acceptors in a network of O—H⋯O hydrogen bonds.

## Related literature

For the crystal structures of Zn^II^ complexes with pyrazine-2,3-dicarboxyl­ato and aqua ligands, see: Richard *et al.* (1974 ▶); Ptasiewicz-Bąk & Leciejewicz (1999 ▶); Gryz *et al.* (2005 ▶). 
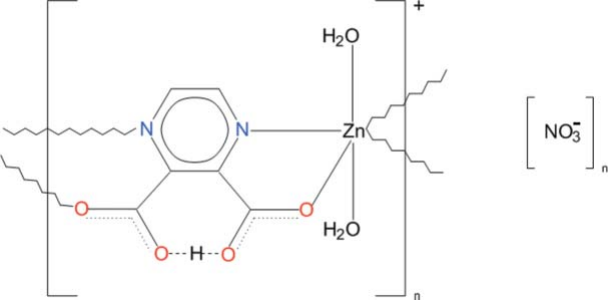


## Experimental

### 

#### Crystal data

[Zn(C_6_H_3_N_2_O_4_)(H_2_O)_2_]NO_3_
*M* *_r_* = 330.52
Monoclinic, 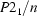

*a* = 8.7431 (17) Å
*b* = 10.867 (2) Å
*c* = 11.412 (2) Å
β = 100.48 (3)°
*V* = 1066.2 (4) Å^3^
*Z* = 4
Mo *K*α radiation
μ = 2.36 mm^−1^
*T* = 293 K
0.20 × 0.19 × 0.15 mm

#### Data collection

Kuma KM-4 four-circle diffractometer
Absorption correction: analytical (*CrysAlis RED*; Oxford Diffraction, 2008 ▶) *T*_min_ = 0.636, *T*_max_ = 0.747
3232 measured reflections
3101 independent reflections
2397 reflections with *I* > 2σ(*I*)
*R*_int_ = 0.012
3 standard reflections every 200 reflections  intensity decay: 1.6%

#### Refinement

*R*[*F* ^2^ > 2σ(*F* ^2^)] = 0.032
*wR*(*F* ^2^) = 0.097
*S* = 1.03
3101 reflections
192 parameters
H atoms treated by a mixture of independent and constrained refinement
Δρ_max_ = 0.81 e Å^−3^
Δρ_min_ = −0.48 e Å^−3^

### 

Data collection: *KM-4 Software* (Kuma, 1996 ▶); cell refinement: *KM-4 Software*; data reduction: *DATAPROC* (Kuma, 2001 ▶); program(s) used to solve structure: *SHELXS97* (Sheldrick, 2008 ▶); program(s) used to refine structure: *SHELXL97* (Sheldrick, 2008 ▶); molecular graphics: *SHELXTL* (Sheldrick, 2008 ▶); software used to prepare material for publication: *SHELXTL*.

## Supplementary Material

Crystal structure: contains datablock(s) I, global. DOI: 10.1107/S1600536811054031/kp2377sup1.cif

Structure factors: contains datablock(s) I. DOI: 10.1107/S1600536811054031/kp2377Isup2.hkl

Additional supplementary materials:  crystallographic information; 3D view; checkCIF report

## Figures and Tables

**Table 1:** Selected bond lengths (Å)

Zn1—O6	2.052 (2)
Zn1—O5	2.069 (2)
Zn1—O4^i^	2.0769 (18)
Zn1—O1	2.0816 (17)
Zn1—N1	2.1663 (18)
Zn1—N2^i^	2.1946 (19)

Symmetry code: (i) 
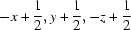
.

**Table 2:** Hydrogen-bond geometry (Å, °)

*D*—H⋯*A*	*D*—H	H⋯*A*	*D*⋯*A*	*D*—H⋯*A*
O6—H61⋯O13^ii^	0.84 (4)	1.90 (4)	2.732 (3)	176 (3)
O5—H52⋯O12^iii^	0.83 (4)	1.88 (4)	2.696 (3)	169 (4)
O6—H62⋯O11^iii^	0.72 (5)	2.04 (5)	2.758 (3)	175 (5)
O5—H51⋯O3^ii^	0.73 (6)	2.37 (5)	2.983 (3)	142 (5)
O5—H51⋯O11^i^	0.73 (6)	2.63 (6)	3.094 (4)	123 (5)
O2—H3⋯O3	1.20 (5)	1.22 (5)	2.404 (2)	170 (4)

Symmetry codes: (i) 
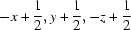
; (ii) 
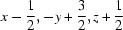
; (iii) 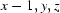.

**Fig. 1.**
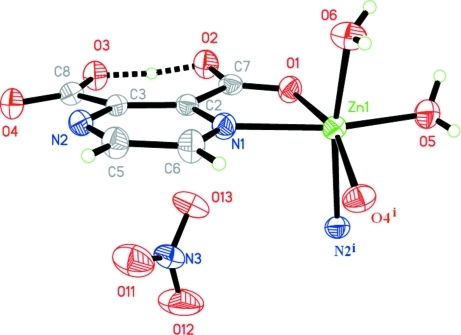
A structural unit of the title compound with atom labelling scheme and 50% probability displacement ellipsoids. Symmetry code: (i) -x + 1/2, y + 1/2,-z + 1/2. An intramolecuular hydrogen bond is shown by dashed lines.

**Fig. 2.**
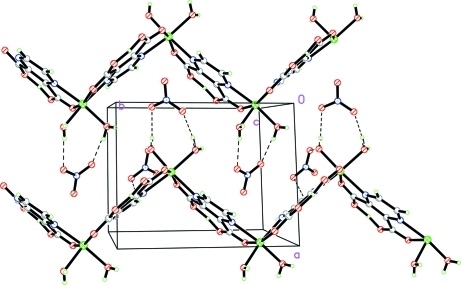
Packing diagram of the structure viewed along the c axis.

## supplementary crystallographic information

### Comment

The structures of three Zn^II^ coordination compounds with pyrazine-2,3-dicarboxylate ligand (2,3-PZDC), each with a different molecular pattern were reported. In the triclinic structure of Zn(2,3-PZDC)(H_2_O)_2_.H_2_O (Richard *et al.*, 1974) molecular ribbons are observed while the monoclinic Zn(2,3-PZDC)(H_2_O)_3_.H_2_O (Ptasiewicz-Bąk & Leciejewicz, 1999) shows a zigzag catenated molecular pattern. The monoclinic structure of (H_3_O)^+^_2_ [Zn(2,3-PZDC)^2-^ is built of catenated doubly layered polyanions with hydronium cations in the interstitials (Gryz *et al.* 2005). The structure of the title compound is composed of zigzag molecular chains propagating along the crystal *b* axis in which Zn^II^ ions are coordinated by *N*,*O* chelating groups of two singly deprotonated ligand molecules; their planes make a dihedral angle of 82.1 (1)° each to the other (Fig. 1). Two water O atoms complete the coordination of the Zn^II^ ion to six, located at the apices of a distorted octahedron. O1, N1, O4^i^ and O5 atoms form its basal plane with r.m.s. of 0.1408 (2) Å.  Zn—O and Zn—N bond lengths are close to those observed in the structures of other Zn complexes with the title ligand (Richard, *et al.*, 1974; Ptasiewicz-Bąk & Leciejewicz, 1999; Gryz, *et al.*, 2005). A pyrazine ring is planar [r.m.s. 0.0146 (2) Å, the carboxylate groups C7/O1/O2 and C8/O3/O4 make with it dihedral angles of 4.8 (1)° and 171.9 (1)°, respectively. One of the carboxylate groups remains protonated and participates in a short, intra-molecular hydrogen bond of 2.404 (2) Å. Consequently, each building unit of the chain shows a singly positive charge which is compensated by a nitrate anion located in the space between chains (Fig. 2). Hydrogen bonds are observed between coordinated water molecules which act as donors and nitrate O atoms as acceptors (Table 2).

### Experimental

Single crystals of the title compound were found incidently in the course of an attempt to obtain dinuclear magnesium-zinc complex with the pyrazine-2,3-dicarboxylate ligand. A solution containing 2 mmols of pyrazine-2,3-dicarboxylic acid dihydrate, 1 mmol of magnesium nitrate dihydrate and a small excess over 1 mmol of zinc nitrate hexahydrate in 100 mL of doubly distilled water was boiled under reflux with stirring for 10 h. After cooling to room temperature, two drops of 95% hydrazine were added to the solution which was left to crystallise. Colourless single-crystal blocks of the title compound and crystals of Zn(H_2_O)_6_(NO_3_)_2_ were found in an unidentified polycrystalline material after evaporation to dryness. The crystals of the title complex were extracted, washed with ethanol and dried in air.

### Refinement

Water hydrogen atoms were located in a difference map and refined isotropically while H atoms attached to pyrazine-ring C atoms were located at calculated positions and treated as riding on the parent atoms with C—H=0.93 Å and *U*_iso_(H)=1.2*U*_eq_(C).

### Crystal data

**Table:**

[Zn(C_6_H_3_N_2_O_4_)(H_2_O)_2_]NO_3_	*F*(000) = 664
*M**_r_* = 330.52	*D*_x_ = 2.059 Mg m^−^^3^
Monoclinic, *P*2_1_/*n*	Mo *K*α radiation, λ = 0.71073 Å
Hall symbol: -P 2yn	Cell parameters from 25 reflections
*a* = 8.7431 (17) Å	θ = 6–15°
*b* = 10.867 (2) Å	µ = 2.36 mm^−^^1^
*c* = 11.412 (2) Å	*T* = 293 K
β = 100.48 (3)°	Blocks, colourless
*V* = 1066.2 (4) Å^3^	0.20 × 0.19 × 0.15 mm
*Z* = 4	

### Data collection

**Table:**

Kuma KM-4 four-circle diffractometer	2397 reflections with *I* > 2σ(*I*)
Radiation source: fine-focus sealed tube	*R*_int_ = 0.012
graphite	θ_max_ = 30.1°, θ_min_ = 2.6°
profile data from ω/2θ scans	*h* = −10→12
Absorption correction: analytical (*CrysAlis RED*; Oxford Diffraction, 2008)	*k* = −15→0
*T*_min_ = 0.636, *T*_max_ = 0.747	*l* = −16→0
3232 measured reflections	3 standard reflections every 200 reflections
3101 independent reflections	intensity decay: 1.6%

### Refinement

**Table:**

Refinement on *F*^2^	Primary atom site location: structure-invariant direct methods
Least-squares matrix: full	Secondary atom site location: difference Fourier map
*R*[*F*^2^ > 2σ(*F*^2^)] = 0.032	Hydrogen site location: inferred from neighbouring sites
*wR*(*F*^2^) = 0.097	H atoms treated by a mixture of independent and constrained refinement
*S* = 1.03	*w* = 1/[σ^2^(*F*_o_^2^) + (0.0564*P*)^2^ + 0.8069*P*] where *P* = (*F*_o_^2^ + 2*F*_c_^2^)/3
3101 reflections	(Δ/σ)_max_ = 0.001
192 parameters	Δρ_max_ = 0.81 e Å^−^^3^
0 restraints	Δρ_min_ = −0.48 e Å^−^^3^

### Special details

**Table:**

Geometry. All e.s.d.'s (except the e.s.d. in the dihedral angle between two l.s. planes) are estimated using the full covariance matrix. The cell e.s.d.'s are taken into account individually in the estimation of e.s.d.'s in distances, angles and torsion angles; correlations between e.s.d.'s in cell parameters are only used when they are defined by crystal symmetry. An approximate (isotropic) treatment of cell e.s.d.'s is used for estimating e.s.d.'s involving l.s. planes.
Refinement. Refinement of *F*^2^ against ALL reflections. The weighted *R*-factor *wR* and goodness of fit *S* are based on *F*^2^, conventional *R*-factors *R* are based on *F*, with *F* set to zero for negative *F*^2^. The threshold expression of *F*^2^ > σ(*F*^2^) is used only for calculating *R*-factors(gt) *etc*. and is not relevant to the choice of reflections for refinement. *R*-factors based on *F*^2^ are statistically about twice as large as those based on *F*, and *R*- factors based on ALL data will be even larger.

### Fractional atomic coordinates and isotropic or equivalent isotropic displacement parameters (Å^2^)

**Table:**

	*x*	*y*	*z*	*U*_iso_*/*U*_eq_	
Zn1	−0.00406 (3)	0.82559 (2)	0.26307 (2)	0.02503 (9)	
C7	0.0503 (2)	0.68275 (19)	0.06020 (18)	0.0235 (4)	
C2	0.1549 (2)	0.61605 (18)	0.16261 (17)	0.0212 (4)	
N1	0.1442 (2)	0.66517 (17)	0.26888 (16)	0.0255 (4)	
C3	0.2546 (2)	0.51569 (19)	0.15702 (18)	0.0214 (4)	
C8	0.3008 (3)	0.45650 (19)	0.04721 (19)	0.0247 (4)	
C6	0.2221 (3)	0.6151 (2)	0.36834 (19)	0.0303 (5)	
H6	0.2153	0.6495	0.4418	0.036*	
O5	−0.1653 (2)	0.96368 (18)	0.2120 (2)	0.0403 (4)	
N2	0.3300 (2)	0.46525 (17)	0.25858 (16)	0.0246 (3)	
O3	0.2334 (2)	0.48857 (16)	−0.05626 (14)	0.0314 (3)	
O4	0.4046 (2)	0.37756 (16)	0.06479 (15)	0.0313 (3)	
O2	0.0421 (2)	0.64513 (17)	−0.04597 (14)	0.0323 (4)	
O1	−0.02615 (19)	0.77131 (15)	0.08585 (14)	0.0295 (3)	
O6	−0.1700 (2)	0.71769 (19)	0.3183 (2)	0.0373 (4)	
C5	0.3133 (3)	0.5120 (2)	0.36293 (19)	0.0294 (4)	
H5	0.3634	0.4752	0.4330	0.035*	
O11	0.5234 (3)	0.7166 (3)	0.2043 (2)	0.0568 (6)	
O12	0.5562 (3)	0.8786 (3)	0.1012 (3)	0.0758 (9)	
O13	0.3449 (2)	0.7771 (2)	0.05949 (19)	0.0463 (5)	
N3	0.4762 (2)	0.7915 (2)	0.1237 (2)	0.0366 (5)	
H61	−0.166 (4)	0.723 (3)	0.392 (3)	0.035 (8)*	
H52	−0.256 (5)	0.946 (4)	0.181 (4)	0.056 (11)*	
H62	−0.251 (6)	0.722 (4)	0.289 (4)	0.081 (16)*	
H51	−0.165 (6)	1.002 (5)	0.265 (5)	0.095 (19)*	
H3	0.143 (5)	0.574 (4)	−0.056 (4)	0.088 (15)*	

### Atomic displacement parameters (Å^2^)

**Table:**

	*U*^11^	*U*^22^	*U*^33^	*U*^12^	*U*^13^	*U*^23^
Zn1	0.02357 (14)	0.02086 (13)	0.02998 (14)	−0.00042 (9)	0.00303 (9)	−0.00081 (9)
C7	0.0212 (9)	0.0232 (9)	0.0241 (9)	−0.0025 (7)	−0.0014 (7)	0.0015 (7)
C2	0.0199 (8)	0.0202 (8)	0.0224 (9)	−0.0013 (7)	0.0010 (7)	0.0010 (7)
N1	0.0253 (8)	0.0249 (9)	0.0249 (8)	0.0031 (7)	0.0006 (7)	−0.0020 (6)
C3	0.0205 (9)	0.0197 (8)	0.0234 (8)	−0.0014 (7)	0.0022 (7)	−0.0003 (7)
C8	0.0260 (10)	0.0236 (9)	0.0246 (9)	−0.0015 (7)	0.0047 (7)	−0.0003 (7)
C6	0.0353 (11)	0.0328 (11)	0.0214 (9)	0.0080 (9)	0.0016 (8)	−0.0015 (8)
O5	0.0317 (10)	0.0276 (9)	0.0589 (13)	0.0062 (7)	0.0010 (9)	−0.0050 (8)
N2	0.0231 (8)	0.0236 (8)	0.0260 (8)	0.0020 (6)	0.0013 (6)	0.0012 (6)
O3	0.0360 (9)	0.0348 (9)	0.0231 (7)	0.0068 (7)	0.0041 (6)	0.0002 (6)
O4	0.0353 (9)	0.0296 (8)	0.0297 (8)	0.0079 (7)	0.0075 (7)	0.0003 (6)
O2	0.0345 (9)	0.0363 (9)	0.0233 (7)	0.0070 (7)	−0.0022 (6)	−0.0015 (6)
O1	0.0291 (8)	0.0264 (8)	0.0295 (8)	0.0060 (6)	−0.0035 (6)	−0.0009 (6)
O6	0.0315 (10)	0.0400 (10)	0.0393 (11)	−0.0078 (8)	0.0041 (8)	0.0034 (8)
C5	0.0318 (11)	0.0309 (11)	0.0236 (9)	0.0057 (9)	−0.0004 (8)	0.0014 (8)
O11	0.0473 (12)	0.0708 (16)	0.0467 (12)	−0.0080 (11)	−0.0060 (9)	0.0132 (11)
O12	0.0526 (15)	0.0659 (17)	0.100 (2)	−0.0293 (13)	−0.0098 (14)	0.0186 (16)
O13	0.0259 (9)	0.0682 (14)	0.0428 (10)	−0.0089 (9)	0.0006 (8)	0.0042 (10)
N3	0.0265 (10)	0.0452 (12)	0.0379 (11)	−0.0069 (9)	0.0052 (8)	−0.0012 (9)

### Geometric parameters (Å, °)

**Table:**

Zn1—O6	2.052 (2)	C6—C5	1.382 (3)
Zn1—O5	2.069 (2)	C6—H6	0.9300
Zn1—O4^i^	2.0769 (18)	O5—H52	0.83 (4)
Zn1—O1	2.0816 (17)	O5—H51	0.73 (6)
Zn1—N1	2.1663 (18)	N2—C5	1.327 (3)
Zn1—N2^i^	2.1946 (19)	N2—Zn1^ii^	2.1947 (19)
C7—O1	1.237 (3)	O3—H3	1.22 (5)
C7—O2	1.268 (3)	O4—Zn1^ii^	2.0769 (18)
C7—C2	1.529 (3)	O2—H3	1.20 (5)
C2—N1	1.344 (3)	O6—H61	0.84 (4)
C2—C3	1.405 (3)	O6—H62	0.72 (5)
N1—C6	1.330 (3)	C5—H5	0.9300
C3—N2	1.342 (3)	O11—N3	1.241 (3)
C3—C8	1.527 (3)	O12—N3	1.230 (3)
C8—O4	1.238 (3)	O13—N3	1.254 (3)
C8—O3	1.269 (3)		
			
O6—Zn1—O5	90.99 (9)	C2—C3—C8	128.54 (18)
O6—Zn1—O4^i^	93.59 (8)	O4—C8—O3	122.9 (2)
O5—Zn1—O4^i^	102.46 (9)	O4—C8—C3	117.00 (19)
O6—Zn1—O1	101.00 (8)	O3—C8—C3	120.06 (19)
O5—Zn1—O1	89.65 (9)	N1—C6—C5	120.2 (2)
O4^i^—Zn1—O1	160.90 (7)	N1—C6—H6	119.9
O6—Zn1—N1	89.06 (8)	C5—C6—H6	119.9
O5—Zn1—N1	164.96 (9)	Zn1—O5—H52	120 (3)
O4^i^—Zn1—N1	92.55 (7)	Zn1—O5—H51	107 (4)
O1—Zn1—N1	75.58 (7)	H52—O5—H51	110 (5)
O6—Zn1—N2^i^	166.71 (8)	C5—N2—C3	120.11 (19)
O5—Zn1—N2^i^	85.27 (8)	C5—N2—Zn1^ii^	123.90 (15)
O4^i^—Zn1—N2^i^	74.86 (7)	C3—N2—Zn1^ii^	115.27 (14)
O1—Zn1—N2^i^	91.74 (7)	C8—O3—H3	114 (2)
N1—Zn1—N2^i^	97.85 (7)	C8—O4—Zn1^ii^	120.67 (15)
O1—C7—O2	122.53 (19)	C7—O2—H3	113 (2)
O1—C7—C2	117.50 (19)	C7—O1—Zn1	119.46 (14)
O2—C7—C2	119.96 (19)	Zn1—O6—H61	112 (2)
N1—C2—C3	119.66 (18)	Zn1—O6—H62	121 (4)
N1—C2—C7	111.79 (18)	H61—O6—H62	108 (4)
C3—C2—C7	128.54 (18)	N2—C5—C6	120.6 (2)
C6—N1—C2	119.93 (19)	N2—C5—H5	119.7
C6—N1—Zn1	124.51 (15)	C6—C5—H5	119.7
C2—N1—Zn1	115.56 (14)	O12—N3—O11	122.2 (2)
N2—C3—C2	119.29 (18)	O12—N3—O13	118.0 (3)
N2—C3—C8	112.05 (18)	O11—N3—O13	119.8 (2)

Symmetry codes: (i) −*x*+1/2, *y*+1/2, −*z*+1/2; (ii) −*x*+1/2, *y*−1/2, −*z*+1/2.

### Hydrogen-bond geometry (Å, °)

**Table:**

*D*—H···*A*	*D*—H	H···*A*	*D*···*A*	*D*—H···*A*
O6—H61···O13^iii^	0.84 (4)	1.90 (4)	2.732 (3)	176 (3)
O5—H52···O12^iv^	0.83 (4)	1.88 (4)	2.696 (3)	169 (4)
O6—H62···O11^iv^	0.72 (5)	2.04 (5)	2.758 (3)	175 (5)
O5—H51···O3^iii^	0.73 (6)	2.37 (5)	2.983 (3)	142 (5)
O5—H51···O11^i^	0.73 (6)	2.63 (6)	3.094 (4)	123 (5)
O2—H3···O3	1.20 (5)	1.22 (5)	2.404 (2)	170 (4)

Symmetry codes: (iii) *x*−1/2, −*y*+3/2, *z*+1/2; (iv) *x*−1, *y*, *z*; (i) −*x*+1/2, *y*+1/2, −*z*+1/2.
